# Neurovascular Coupling in Type 2 Diabetes With Cognitive Decline. A Narrative Review of Neuroimaging Findings and Their Pathophysiological Implications

**DOI:** 10.3389/fendo.2022.874007

**Published:** 2022-07-04

**Authors:** Mads C. J. Barloese, Christian Bauer, Esben Thade Petersen, Christian Stevns Hansen, Sten Madsbad, Hartwig Roman Siebner

**Affiliations:** ^1^Danish Research Centre for Magnetic Resonance, Centre for Functional and Diagnostic Imaging and Research, Copenhagen University Hospital - Amager and Hvidovre, Copenhagen, Denmark; ^2^Department of Clinical Physiology and Nuclear Imaging, Center for Functional and Diagnostic Imaging, Copenhagen University Hospital - Amager and Hvidovre, Copenhagen, Denmark; ^3^Radiography, Department of Technology, University College Copenhagen, Copenhagen, Denmark; ^4^Center for Magnetic Resonance, Department of Electrical Engineering, Technical University of Denmark, Lyngby, Denmark; ^5^Steno Diabetes Center Copenhagen, Herlev, Denmark; ^6^Department of Endocrinology, Copenhagen University Hospital - Amager and Hvidovre, Copenhagen, Denmark; ^7^Department of Clinical Medicine, Faculty of Health and Medical Sciences, University of Copenhagen, Copenhagen, Denmark; ^8^Department of Neurology, Copenhagen University Hospital - Bispebjerg and Fredriksberg, Copenhagen, Denmark

**Keywords:** type 2 diabetes (T2D), cognitive decline, neurovascular coupling (NVC), neuroimaging, alzheimer’s disease

## Abstract

Type 2 diabetes causes substantial long-term damage in several organs including the brain. Cognitive decline is receiving increased attention as diabetes has been established as an independent risk factor along with the identification of several other pathophysiological mechanisms. Early detection of detrimental changes in cerebral blood flow regulation may represent a useful clinical marker for development of cognitive decline for at-risk persons. Technically, reliable evaluation of neurovascular coupling is possible with several caveats but needs further development before it is clinically convenient. Different modalities including ultrasound, positron emission tomography and magnetic resonance are used preclinically to shed light on the many influences on vascular supply to the brain. In this narrative review, we focus on the complex link between type 2 diabetes, cognition, and neurovascular coupling and discuss how the disease-related pathology changes neurovascular coupling in the brain from the organ to the cellular level. Different modalities and their respective pitfalls are covered, and future directions suggested.

## Introduction

Driven by changing demographics and lifestyle factors, diabetes mellitus will affect half a billion people worldwide within a few decades, with severe economic and personal consequence (1). A recent study estimated the projected number of adults with diagnosed diabetes to increase from 22 million to 61 million in 2060 in The United States (2). In the setting of strained health care provision with exigent, concurrent demands for efficiency and quality, providing optimum care and preventing comorbidity in diabetes is challenging.

Type 2 diabetes (T2DM) is by far is the most prevalent of the two subtypes of diabetes, making up 90-95% of cases (3). It develops as a result of impaired beta-cell function in combination with insulin resistance in the tissues. The resulting hyperglycemia, along with dyslipidaemia and hypertension, has detrimental effects on many organ systems (4). T2DM causes substantial long-term morbidity with late diabetic complications from the eyes, kidney and nervous system as well as increased risk of arteriosclerosis (5). One clinical manifestation which is receiving increasing attention is cognitive impairment or so-called diabetic “cogno-pathy” (6). T2DM has been identified as an independent risk factor for cognitive decline evolving into manifest Alzheimer’s disease (AD) (7–9). T2DM patients with elevated HbA1c levels (10), and intriguingly non-diabetics with acute elevated blood glucose levels (11) as well as cognitively intact adults with pre-diabetes (12), have decreased metabolism in brain regions characteristic for AD. Furthermore, possible effects on AD of anti-diabetic drugs such as pioglitazone, which reduce insulin resistance or the Glucagon-Like Polypeptide-1 receptor agonist (GLP-1 RA), with an effect on low-grade inflammation, are being investigated (13). These findings motivate the search for biomarkers that are sensitive to early functional brain changes on which prognosis and intervention can be based.

Decades of intense research, including studies on patients with stroke or headache, have advanced our understanding of the physiological regulation of the brain’s blood supply and its pathophysiological relevance (14). However, an obstacle in understanding the physiology is that with decreasing vessel diameter, it becomes increasingly difficult to probe regulatory mechanisms and study the tight functional interactions between vessels, neurons and glia (15).

The concept of neurovascular coupling (NVC) describes a cellular mechanism by which neuronal activation induces concurrent local increases in cerebral blood flow (CBF). These local increases of blood supply are critical to brain function, and impaired NVC may play an early role in triggering cognitive dysfunction in T2DM (16). In diabetes, cognitive ability is influenced by multiple factors at the systemic level, such as the degree of extra- and intra-cranial atherosclerosis, dysfunction of glymphatic tissue clearance (17), and cellular dysfunction, such as altered receptor expression. The identification of neurovascular abnormalities that are attributable to diabetes and precede structural and clinical changes, holds the potential to guide personalized, preventive interventions (18).

**Figure 1:**
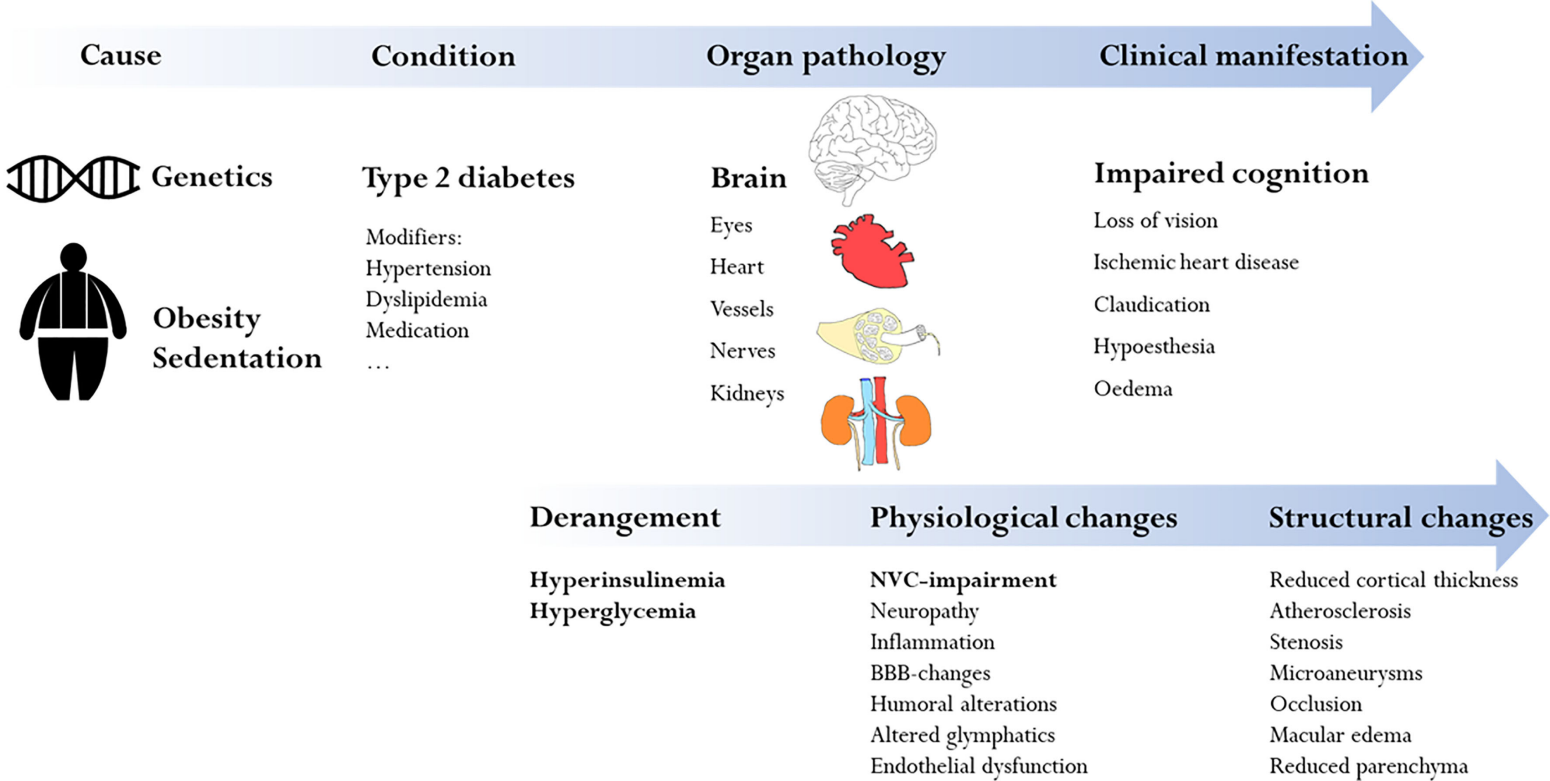
Overview of the complex and multifactorial mechanisms which lead to impaired cognition in type 2 diabetes. The focus of this review is the specific diabetic influence on neurovascular coupling and how this leads to impaired cognition (bold in the figure).

In this narrative review, we focus on how NVC is impaired by T2DM and how we can measure T2DM-related neurovascular dysfunction in humans (**Figure 1**). We will first provide a brief introduction to diabetes, cognitive decline, and the neurovascular architecture. We will then discuss current concepts of how diabetes affects NVC and in what way this relates to cognitive decline. In the last section, we will review commonly employed methodology that has contributed to our understanding of NVC and its alteration in T2DM, with a focus on magnetic resonance imaging (MRI). It bears mentioning that while a detailed understanding of individual cellular mechanisms is within reach in bench models, translating and relating this to clinical or pre-clinical observations is not always possible.

## Cognitive Decline in the Setting of Diabetes

The relative general prevalence of dementia in individuals aged over 60 years is 6-7% (19). Diabetes confers a 1.3 to 1.9-fold increased risk of cognitive impairment, but even pre-diabetes and diabetes-associated biochemical changes (fasting glucose, postload glucose, glycosylated hemoglobin, insulin) predict cognitive decline (20). Also, diabetes is one of nine potentially modifiable risk factors modelled by the 2017 Lancet Commission on dementia prevention, intervention and care (21). Cognitive decline encompasses subjective cognitive decline [reviewed in (22)], mild cognitive impairment and manifest dementia with AD being the most frequent underlying disease (**Table 1**). AD can be divided into AD-pathophysiological process (AD-P), which precedes the clinical phase (AD-C), and may include patients with cognitive impairment due to AD-P before clinical onset (25). The pathology behind AD is complex, involving neuroinflammation and accumulation of β-amyloid and tau protein leading to neuronal death and atrophy in specific cortical areas (26). Thus, risk factors include T2DM and genetics, among others, but the single most relevant is age (27).

**Table 1:** Operational definitions of cognitive decline.

Diabetic “cognopathy” (6)	Research term referring to cognitive impairment (e.g., memory impairment, reduced psychomotor speed, affected executive function, verbal fluency and attention) that is attributable to diabetes mellitus, typically associated with functional and structural changes in the brain
Subjective cognitive decline (23)	Self-experienced persistent decline in cognitive capacity in comparison with a previously normal status and unrelated to an acute event. Normal age-, gender-, and education-adjusted performance on standardized cognitive tests, which are used to classify MCI or prodromal AD. 1 and 2 must be present Exclusion criteria: Mild cognitive impairment, prodromal AD, or dementia Can be explained by a psychiatric or neurologic disease, medical disorder, medication, or substance use
Mild cognitive impairment (MCI) (24)	Measurable cognitive impairment without effect on activities of daily living. This diagnostic label is applied if there is no disease to which MCI can be attributed. Term of exclusion.
Alzheimer’s disease (AD) (24)	Progressive cognitive decline (i.e., impaired memory) Preserved consciousness Disrupted emotional control Duration of at least 6 months *In-vivo* markers of Alzheimer’s pathology: Corticospinal fluid (CSF): amyloid β, total tau, and phospho-tau Positron emission tomography (PET): Regional accumulation of amyloid and tau tracers, reduced mid-temporal and mid-parietal glucose metabolism Structural magnetic resonance imaging (MRI): Atrophy of medial temporal lobe, medial parietal cortex

## Definition of Cognitive Decline

**Figure 2:**
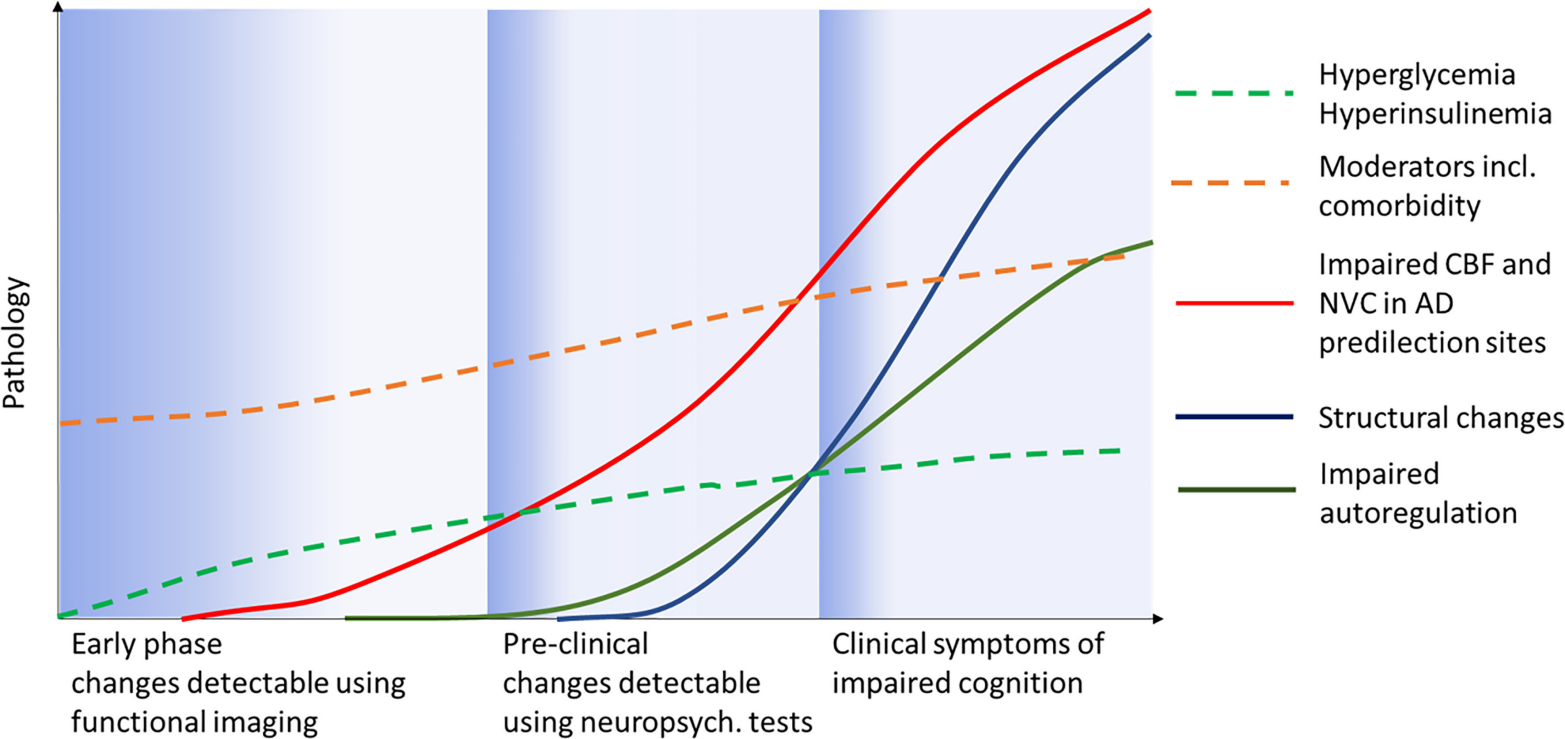
Possible scenario for clinical progression (x-axis) from pre-diabetes to manifest clinical diabetic “cognopathy”. Moderators and comorbidity represent all diseases and factors present before and after T2DM onset such as sleep apnea, obesity, hypertension etc. Adapted from Jessen and colleagues (27).

The mechanism by which diabetes induces cognopathy was originally ascribed to vascular changes but this model is too simple as multiple vascular and non-vascular processes act in concert (28) (**Figure 2**). It has been demonstrated that cognition can be affected by hyperglycemia, changes to insulin secretion and sensitivity, T2DM complications, comorbidity as well as certain medications. New findings also show that the diabetes-induced metabolic milieu is specifically conducive to AD-P processes with greater β-amyloid plaque and tau deposition, advanced glycation end products and activated microglia in diabetic AD compared to non-diabetic AD (29, 30). At the pathophysiological level, multiple mechanisms have been implicated, including impaired NVC, a malfunction of cerebrovascular autoregulation (31) and glucose transport (32), neuroinflammation (33) and insulin resistance (9). Of note, insulin crosses the blood-brain barrier (BBB) and acts as a neuropeptide in the central nervous system having distinct neuromodulatory effects on key brain structures (34). Animal studies have shown that insulin targets astrocytes (35) and has trophic actions promoting synapse growth, neuron maintenance and repair as well as improving hippocampal synaptic plasticity (36). These findings indicate an intimate relationship between diabetes and AD which led to the proposal to consider AD as “type 3 diabetes” (37), and prompted clinical trials testing the efficacy of anti-T2DM drugs such as liraglutide (38), thiazolidinediones (39), intranasal insulin (40) and metformin (41) in AD with positive preliminary results (13).

## Autoregulation and Neurovascular Coupling

Precise spatial and temporal titration of CBF supply is critical to brain function. Cerebrovascular autoregulation stabilizes regional cerebral blood flow by sheltering it from fluctuations in systemic perfusion pressure. This mechanism is partly intrinsic to smooth muscle cells at the pial arteriole/parenchymal section, which relax and contract according to the transmural pressure, referred to as the myogenic response (42). NVC refers to a separate mechanism ensuring that perfusion is adjusted to the neuro-metabolic demands at the cellular level. NVC can be thought of as a variable resistor that works at the level of parenchymal arterioles “in series” with, and at the level of pial vessels, “in parallel” with cerebral autoregulation.

### Innervation of Cerebral Vasculature

The density and nature of regional innervation of the cerebral vasculature varies considerably depending on the lobe and segment of the vascular tree. Based on fiber origin, the innervation can be divided into intrinsic and extrinsic projections. The perivascular nerves of the adventitia of pial arteries and arterioles are external, while microvessels along with interneurons and astrocytes receive intrinsic innervation (43).

The intrinsic neurovascular supply originates from the locus coeruleus (noradrenaline), the raphe nuclei (serotonin), the ventral tegmental area and the nucleus basalis (acetylcholine). These projections from the basal nuclei innervate the vasculature and the cells of the neurovascular unit, particularly astrocytes, without leaving the brain (43). Although its role in NVC is poorly understood, relevant receptors are present on the involved cells and CBF changes can be invoked in response to stimulation of the mentioned nuclei (43).

The extrinsic neurovascular supply system is composed of sympathetic, parasympathetic and sensory nerve fibers (44) running in the adventitia of pial arteries and arterioles (45). These fibers, which predominantly originate in the superior cervical ganglion (sympathetic), otic and sphenopalatine ganglia (parasympathetic) and the trigeminal ganglion (sensory), follow various paths including the ethmoidal nerve to re-enter the cranial cavity. These systems are controlled by brainstem and mesencephalic circuits and seemingly have no major function in autoregulation during physiological conditions (46). However, in certain states such as hypercapnia-induced vasodilation or chronic conditions they do exert influence (47). In hypertension, sympathetic innervation extends autoregulation to higher pressures and may protect the brain against pressure surges whereas sensory innervation may serve a protective role restoring vessel tone after constriction (48, 49).

### The Neurovascular Unit

The neurovascular unit (NVU) consists of a set of cells that intimately interact to enable a temporal and spatial NVC and secures that local blood supply is rapidly aligned to moment-to-moment changes in regional neural activity and associated fluctuations in metabolic demand and waste production (45). The cells involved are neurons (pyramidal cells and interneurons), astrocytes, smooth muscle cells, endothelial cells, and pericytes (45). Depending on the ongoing level of regional activity, neurons and astrocytes release vasodilatory factors that act directly on the perivascular cells to induce vasodilation and increase local arterial blood supply.

**Figure 3:**
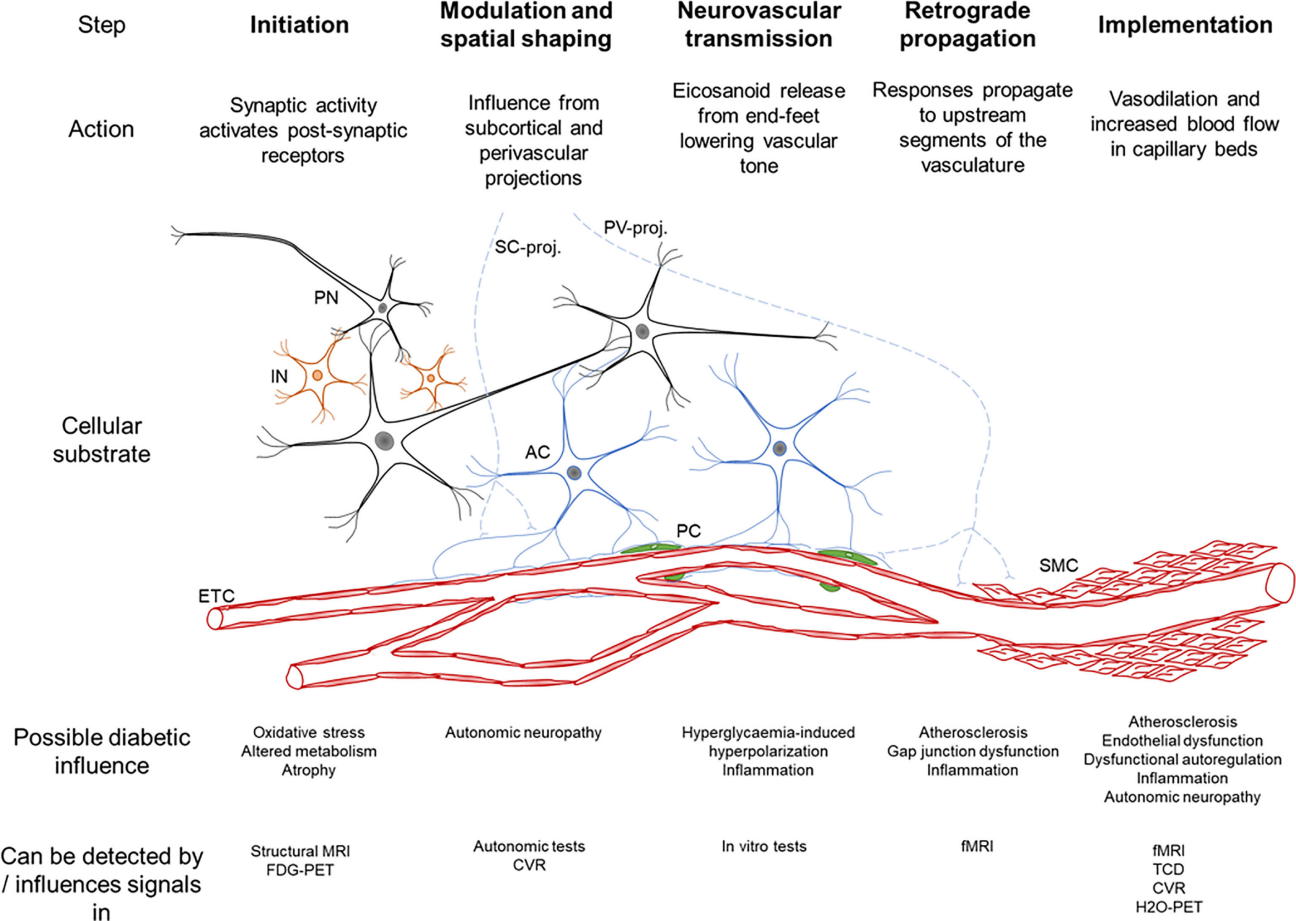
Schematic summary of the neurovascular coupling cascade (46). The cellular substrate for each step is seen, PN – principal neuron, IN – interneuron, AC – astrocyte, PC – pericyte, SMC – smooth muscle cell, ETC – endothelial cells, SC-proj. – subcortical projections from locus coeruleus, basal forebrain, raphe nuclei, PV-proj. – perivascular projections from cranial autonomic ganglia. The bottom rows describe the possible influence of diabetes on each step and how this has been detected.

NVC involves five consecutive steps: Initiation, modulation and spatial shaping, neurovascular transmission, retrograde propagation and implementation (**Figure 3**) (45). It is noteworthy that influences at the arteriolar and capillary level differ since arterioles, as opposed to capillaries, are not only subject to locally mediated vasodilation in response to neuronal activation, but also to retrograde propagation from capillaries which also reaches pial arteries (45). NVC employs both feed-forward (glutamate receptors, Ca^2+^, nitric oxide (NO), eicosanoids) and feed-backward (adenosine, lactate and CO_2_) signaling mechanisms, and the many messengers involved provide redundancy and condition-dependent signaling reflecting the previous and current state of the system (50).

*Neurons. About* eighty percent of the brain’s energy expenditure is attributed to the generation of action potentials in neurons, maintenance of ion-concentrations and postsynaptic effects (51). Any perturbation of cellular supply compromises the signaling function of neurons. To secure sufficient neuronal blood supply, neurons directly control their own homeostatic environment through glutamate actions on N-methyl-D-aspartate (NMDA)-receptors which, *via* downstream signaling and nitric oxide synthase (NOS)-activation with subsequent increase in NO-synthesis, induces vasodilation (52). The same glutamate signal also activates receptors on neighboring astrocytes.

Although most cortical neurons in the cortex are glutamatergic pyramidal cells, gabaergic inhibitory interneurons are also relevant. The gabaergic interneurons project to microvessels influencing the release of NO, prostanoids, endothelin among others (53). The relative contribution of pyramidal cells or interneurons to NVC likely depends on multiple factors including location and the neuronal circuit in question and requires further investigation (54).

*Astrocytes* are glial cells of key importance to NVC by virtue of their perisynaptic and endfeet processes extending from their soma (55, 56). They exert differential control according to the metabolic state of the tissue through constriction and dilation control pathways (57). *In vitro* studies have cast light on the multiple factors influencing the balance between these vasoconstrictors and -dilators including previous vascular tone (58), NO (59), O_2_, lactate and adenosine (57). As the primary neurotransmitter, glutamate in itself activates specific astrocyte group 1 metabotropic glutamate receptors (mGluR) leading to increasing calcium concentrations which in turn forces release of vasoactive substances (dilator and constrictor eicosanoid gliotransmitters) from astrocyte endfeet proximate to the smooth muscle cells (SMC) lining the vessels (60). Phospholipase A_2_ activation induces release of arachidonic acid which is converted to prostaglandins (for example PGE_2_, PGI_2_) and epoxyeicosatrienoic acids (EETs). These reduce vascular tone *via* prostaglandin receptor activation and TRPV4- and BK_Ca_-channels. Detrimental increase of vascular tone happens when arachidonic acid is converted to 20-hydroxyeicosatetraenoic acid (20-HETE) which may occur pathologically (61). It has been observed that under physiological conditions simultaneous activation of both neurons and astrocytes induces a 4-fold increase in local CBF than the increase in ATP (60 vs 15%) which is indirectly supportive of a feed-forward mechanism (62).

*Smooth muscle cells, pericytes and endothelium.* Smooth muscle cells and pericytes make up the vasomotor apparatus of the NVU (45). These cells ultimately determine vascular tone on the basis of neuronal, astrocytic and possibly intrinsic system influence (43). In cerebral capillaries, pericytes replace smooth muscle cells and also serve to maintain structure and BBB (63). Pericytes likely exist in both contractile and non-contractile variations (64) and are interspersed at regular intervals along these vessels (65). Although they have been shown to dilate and constrict in response to various stimuli (66), including amyloid β (67) and during mild CO_2_-challenges (68), results are divergent and their contribution to NVC is debated (69, 70). Recent results suggest a substantial but slow regulation of capillary diameter by pericytes, again introducing a serial layer of control of tissue perfusion (68).

The endothelium itself possesses strong intercellular vasodilators including NO and endothelin and has gap junctions with vasomotor cells ensuring retrograde propagation (45). The labile NO itself seems to exert influence dependent on its concentration dynamics but *in vivo* studies are sparse (71). Recent research has also identified a caveolae-mediated pathway in arteriolar endothelial cells as a major mechanism of neurovascular coupling. Caveolae are invaginations of the plasma membrane that are specifically abundant in arteriolar endothelial cells and mediate NVC independently of endothelial NOS (72). It has been hypothesized that caveolae in the arteriolar endothelial cells may serve as local clusters for ion channels and receptors that convey vasodilatory signals to adjacent smooth muscle cells (72). A recent study in mice found that arteriolar endothelial cells are unique in that they possess abundant caveolae (72). It seems this caveola-specific function in NVC acts independently of the NOS-pathway described above as partial ablation of either NOS or caveolae both partially impair NVC. Ablation of both pathways induces complete decoupling (72).

## The Influence of Diabetes on Cerebral Blood Flow

While it is now generally accepted that diabetes mellitus affects NVC, it remains a challenge to dissect the contributions of chronic hyperglycemia, dysinsulinemia and other modifiers such as hypertension, aging and still other variables (**Figure 1**, also, see section on investigations in humans below). This has been exemplified in the attempt to disentangle diabetic and pre-diabetic vascular complications from the associated (sub-) clinical manifestations (73).

Hyperglycemia itself has acute and chronic adverse effects on NVC. In humans, acute hyperglycemia reduces light-flicker induced vasodilation of retinal arteries (74). Such impairment has been confirmed in animal models pointing to hyperpolarization at the gliovascular interface as the possible mechanism (75–77). This would occur when neuronal activation and subsequent Ca-increase in the endfeet result in potassium-increase and Kir-channel activation in adjacent smooth muscle cells (76). Implicating the NOS-pathway, administration of sodium nitroprusside (an NO donor), seems effective in ameliorating such hyperglycemia-induced decoupling (77). Whether manipulating NO-pathways in humans represents a valid therapeutic avenue remains to be seen.

Increased glucose concentrations also induce oxidative stress and compromise the function of gap junctions of *in vitro* astrocytes (78). Oxidative stress represents an important pathogenetic factor that is shared between T2DM and AD, contributing to endothelial and microvascular dysfunction with neurovascular uncoupling in T2DM (79) and increased amyloid-β deposition in AD (80). Hyperglycemia has also been shown to increase tau phosphorylation in hippocampal neurons of diabetic rats, involving a reduced expression of caveolin-1, the essential structure protein of caveolae, mentioned above, and activation of the mTOR/S6K signaling pathway (81). Namely caveolae represent a recently discovered research target with particular relevance for NVC.

Transcranial doppler (TCD) and functional MRI studies dominate the available clinical knowledge but findings are not entirely congruent (16). Phase-contrast MR measurements did not identify global CBF differences between T2DM patients and controls although it did correlate with cognitive ability (82). Also, global CBF did not predict changes over time suggesting that deteriorating cerebral perfusion does not drive cognitive decline (83). Regional blood flow assessed using arterial spin-labeling (ASL)-MRI confirmed these findings to a degree with comparable CBF reductions in patients with T2DM with subjective cognitive decline, vascular dementia and AD compared to controls (84). However, impaired glycemic control was related to reduced CBF hinting at a possible specific diabetes mechanism.

Overall, these results indicate that CBF-changes and cognitive decline in T2DM are determined by tertiary risk factors and not a particular T2DM pathology. Conversely, other studies have found compelling evidence for T2DM specific changes. Cerebral hypoperfusion has been shown with ASL-MRI in individuals with T2DM (85–87). While the magnitude of hypoperfusion varies, it correlates with cognitive declineClick or tap here to enter text.. In one study, an interaction between hypoperfusion and hypertension suggests that increased blood pressure may precipitate the CBF-decrease, possibly involving compromised autoregulation (87). In these populations, brain atrophy was comparable to controls suggesting that altered perfusion precedes structural changes. Another ASL-study investigated healthy controls, patients with insulin resistance (but not diabetes) and T2DM patients (88). Here, CBF fluctuated with spontaneous end-tidal CO_2_ indicating intact cerebrovascular reactivity (CVR) in manifest T2DM and healthy controls, but not in unmedicated patients with insulin resistance. This was speculated to be attributable to glucose-lowering medications, statins and antihypertensives, thought to increase NVC in the diabetics but not the unmedicated insulin resistance group.

Regional low-grade neuroinflammation represents another overlap between neurodegeneration and diabetes (89)It is likely that the mechanism leading to CNS insulin resistance in Alzheimer’s disease, diabetes and obesity in general is the same and involves such persisting low-grade neuroinflammation. This is likely caused by recruitment of macrophages and secretion of an “inflammatory soup” with cytokines such as TNF-α, IL-1β and IL-6 (90). The initiation of the inflammation cascade can be ascribed to various elements including toxic metabolites, ischemia, infection, trauma. This is a difficult research target as it likely occurs over several years and before symptom onset. Neuroinflammation may induce exaggerated vasoconstriction and diminished vascular reactivity (89). In humans, a prospective ASL-study in T2DM patients supported this view (91). Using CO_2_-rebreathing to assess CVR, prospective rCBF measurements showed diminished reactivity after just two years which was associated with a decrease in cognitive ability (91). This T2DM group also had higher inflammation markers, the levels of which corresponded with decreases in vasoreactivity independently of glycemic and blood pressure control.

While ASL provides measures of regional blood flow, functional MRI (fMRI) maps regional changes in the blood oxygen level-dependent (BOLD) signal (92). The regional BOLD signal, with some caveats (see below), can be used to specifically assess regional NVC at the tissue level (93). BOLD fMRI in early T2DM revealed changes in the hemodynamic response function were observed indicating deterioration of NVC (94). Using similar methods in a breath-hold paradigm, Tschistiakova and colleagues showed that T2DM patients with hypertension had decreased CVR and cortical thickness compared to patients with only hypertension, again suggesting a specific T2DM effect on NVC (95). Hu and colleagues pioneered co-analyzing resting state fMRI and ASL data, an elaboration upon methods previously applied in schizophrenia and depression, to develop specific NVC biomarkers (96, 97). They found that several of these hybrid markers were decreased regionally in T2DM patients without cognitive impairment which might identify patients where early intervention would arrest a pathological cerebrovascular trajectory.

Astrocyte involvement in maintaining BBB also deserves mention in this context. White matter lesions (WML) are associated with increased risk of dementia and cognitive decline as well as stroke (98). Their cause is disputed but may relate to BBB dysfunction (99). In T2DM, cognitive decline is associated with WMLs, atrophy, infarcts and HbA_1c_ and BBB permeability may be increased in these patients (100, 101). Thus, there is an indirect connection between WMLs and BBB disruption in the setting of T2DM with cognitive decline, however, findings are not homogenous and further studies are needed as the specificity of these changes is debatable (102).

### Effect of Antidiabetic Treatment on NVC

Therapeutic manipulation of impaired NVC is in its infancy. Aside from improving vascular health, other interventions may protect against cognitive decline (103). Resveratrol may acutely enhance cerebrovascular responsiveness, as measured by TCD, and possibly also clinically measures of cognition but findings need to be reproduced (104, 105). In diabetic mice, empagliflozin, a sodium glucose transporter inhibitor, ameliorated detrimental structural changes in the NVU (106), possibly through a specific action on astrocyte foot process detachment (107). Other drugs may have detrimental effects, for example, non-steroidal anti-inflammatory drugs including indomethacin and naproxen have been shown to attenuate NVC (108). Semaglutide, a long-acting GLP-1 analogue, which is very effective in T2DM, is entering phase 3 development for the indication of Alzheimer’s disease. Both clinical and preclinical studies have shown promising results with regards to this drug’s effect on cognitive decline (109, 110). Several mechanisms have been suggested and it is particularly interesting that these drugs may have anti-inflammatory properties (111).

## Investigating Neurovascular Coupling in Humans

In humans, NVC is studied non-invasively using MRI, positron emission tomography (PET) and TCD. NVC is typically studied as the regional or global response in blood flow to various forms of stimulation. In the following section, we discuss the strengths and weaknesses of relevant modalities to probe NVC.

### Transcranial Doppler Ultrasound

TCD is highly accessible, non-invasive, safe and provides measurements in real-time with high temporal resolution (112). Blood flow velocities in the major arteries are measured as regional CBF surrogates and evoked changes are typically in the range of 10-20% in the posterior and 5-8% in the middle cerebral artery (50, 113). Vessel diameter changes with blood gas composition and during hypercapnia, metabolic regulation of the NVC is reduced during passive flexion of the arm (114). Consequently, changes are reliable only if vessel diameter is unchanged which may be fair to assume when looking at rapid responses (115). However, end-tidal CO_2_ does fluctuate on a breath-by-breath basis and CBF measurements by TCD may be falsely lowered during hypoxia and hypercapnia (116). Also, reliable measurements require user experience and while being highly accessible, portable and non-invasive this method’s sensitivity and reproducibility may be lower and is highly user-dependent.

### Positron Emission Tomography and Single-Photon Emission Computed Tomography

The tracer employed defines the usefulness of PET to study biological processes. With regards to studying metabolism and blood-flow ^18^F-fluro-deoxy-glucose (FDG) and ^15^O-H_2_O water are gold standard. Amyloid tracers are available and used clinically in the diagnosis of AD. Cellular FDG-uptake is representative of glucose metabolism in the 20-30 minutes following tracer injection with a theoretical spatial resolution of around 4-6 mm. While the time resolution is low, this method allows investigation of *neurometabolic* coupling (change in cerebral metabolic rate vs. blood flow (ΔCMR_Glu_/ΔCBF)). NVC *per se* is not always defined in the same manner and FDG-PET as well as calibrated BOLD (see below) allows for more stringent measurement of the neuronally-induced change in CBF at an arteriolar and capillary level whether this is defined as neuro-metabolic or neurovascular coupling.

The freely diffusible ^15^O-H_2_O, as detected by PET, is the gold standard for minimally-invasive measurement of blood flow and can detect transient phenomena of around 30 sec. Academically, it has been discussed whether glucose metabolism is an accurate proxy for neuronal activation. However, at least in health, perfusion in the CNS is closely coupled to metabolism. Indeed, the case has been made that regional CBF increases during activation is driven primarily by coupling to glucose metabolism whereas oxygen consumption increases are less pronounced (117). The PET-modality can be combined with CT or MR to give hybrid measurements of CBF and brain anatomy and function. The major drawbacks are the use of ionizing radiation and limited availability.

### Magnetic Resonance Imaging

Compared to PET, fMRI has the advantage of excellent spatial resolution and not using ionizing radiation although sometimes contrast agents are required. Several MRI methods are relevant in the study of NVC including BOLD, ASL and phase-contrast fMRI. Gadolinium has been used to evaluate the intactness of the BBB. Cardiovascular reactivity has been assessed using ASL and BOLD fMRI during breathing of CO_2_-enriched gas, breath-holding or rebreathing.

#### Arterial Spin Labeling

ASL quantitates regional CBF without use of contrast or radiation. The method labels blood water molecules in a slab and tracks them circulating the brain. Clinically, ASL can distinguish normal brains from AD (118) and in the research setting it directly allows evaluation of NVC during a neurostimulation paradigm. Discussion is ongoing whether regional CBF, measured by ASL, correlates with oxygen and glucose consumption. However, FDG-PET and ASL-MRI correlate in CBF and the cerebral metabolic rate of oxygen (CMR_O2_) and glucose metabolism (r=0.54, p<0.0001 and r=0.31, p=0.005 respectively) indicating that ASL-CBF reflects oxygen and glucose metabolism (119, 120). Combining fMRI and ASL, functional and CBF maps can be assessed together and may provide more specific markers for neurovascular decoupling (96, 97).

#### Blood Oxygen Level Dependent MRI

**Figure 4:**
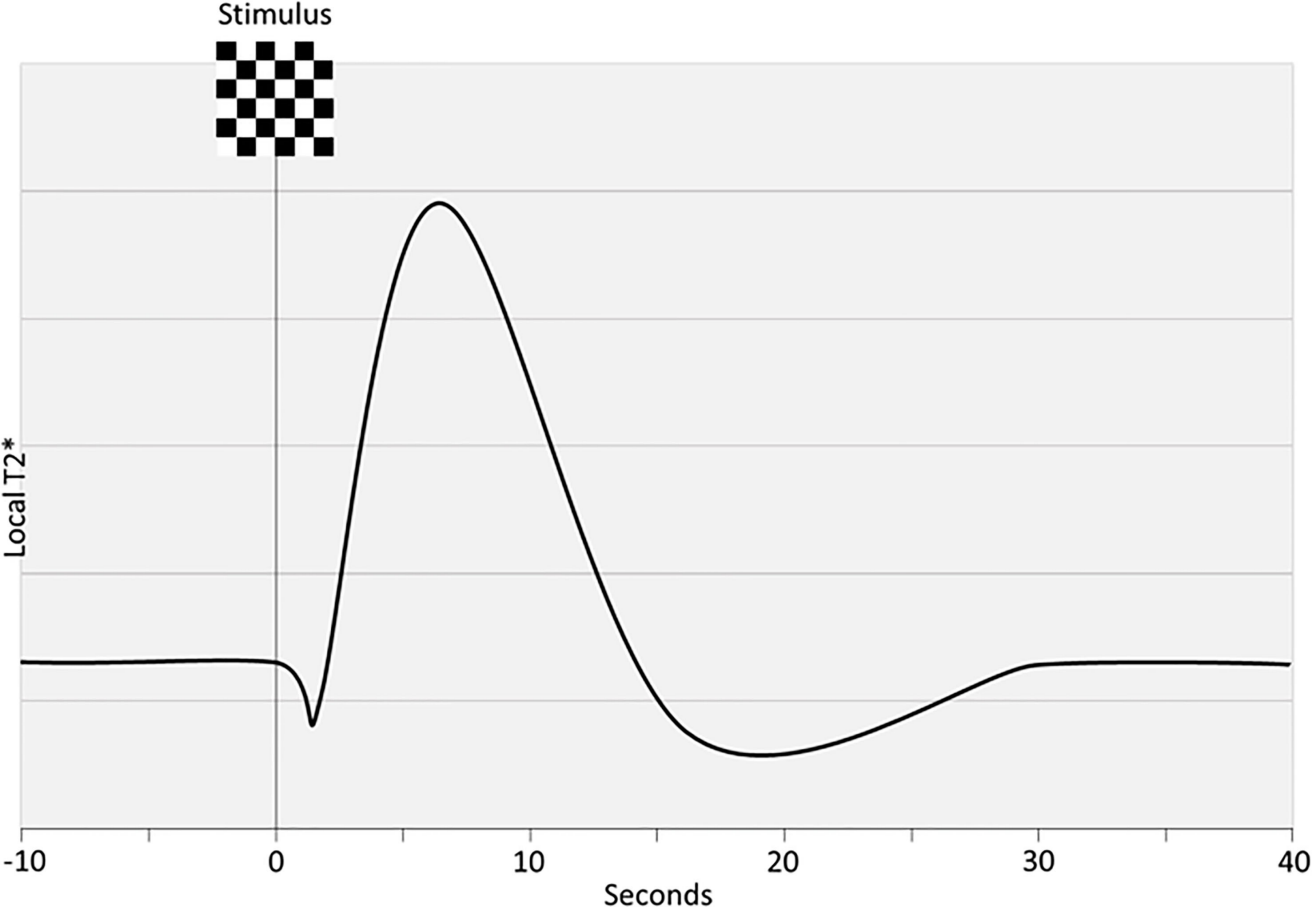
Idealized BOLD response. Steady-state is disrupted by a stimulus which results in an initial dip, an overshoot and lastly an undershoot before steady-state is re-acquired. The rising curve reflects an increase in magnetic field uniformity due to washout of paramagnetic deoxyhemoglobin.

BOLD fMRI can capture the regional vascular response to neuronal activation with high temporal and spatial resolution in heavily T2*-weighted sequences (**Figure 4**). To use this signal as a measure for NVC requires factoring in dynamics of CBF, volume and cerebral metabolic rate for oxygen (CMRO_2_). It is a proxy for neuronal activation and its validity relies on intact physiological cascades. The BOLD-signal reflects the uniformity of the magnetic field in response to paramagnetic deoxyhemoglobin washout from the capillary bed. Thus, deoxyhemoglobin can be thought of as an endogenous contrast agent. With no NVC, neuronal activity would result in increased deoxyhemoglobin and a decreased BOLD signal. However, NVC induces an overcompensating flow increase leading to a relative *decrease* of deoxyhemoglobin and an *increase* in the BOLD signal. That NVC is likely initially driven by glutamate-release and not O_2_ consumption, means that theoretically BOLD is a measure of the intactness of NVC as induced by synaptic activity (15). Thus, reduced BOLD signals can indicate decreased neuronal activation or dysfunctional NVC at some point in the cascade. To mitigate other influences BOLD signals can be evaluated in conjunction with ASL and a vascular challenge, a combination also called calibrated BOLD (121). This combination allows disentanglement of the neuro-metabolic response from the vascular CBF response to a stimulus, that is ΔCMRO_2_/ΔCBF. Other methods have been used to disentangle vascular and neural factors including normalization to baseline CBF or CVR; comparison with other neuronal markers such as electro-encephalogram, magnetoencephalography and PET; and statistical modelling (122). Event-related approaches and performance-matched stimuli are likely preferable in group comparisons (94, 96, 97).

#### Phase-Contrast MRI

Phase contrast MRI has been used to assess blood velocity or bulk flow in supplying vessels and is based on the principle that hydrogen nuclei moving through a magnetic field gradient will acquire a velocity-dependent phase shift. Together with brain volume, acquired from a structural scan, CBF per ml brain tissue per minute can be obtained and corrected for brain tissue density. The method provides absolute measures of global blood flow with a high temporal resolution and without requiring a contrast agent.

#### Dynamic Contrast-Enhanced MRI

Lastly, pericyte control of microcirculation may be affected and a measure for their function may be capillary transit time heterogeneity which can be measured with dynamic contrast-enhanced MRI (123). The biological basis is an increase in transit time heterogeneity following neuronal activity and consequent capillary recruitment. Theoretically, compromised regulation of this capillary dilation, as would be expected to be present in T2DM-mediated neurovascular uncoupling, would manifest as increased capillary transit time heterogeneity. This has not yet been investigated in T2DM to our knowledge.

#### Technical Considerations

Technical limitations need to be considered when measuring regional cerebral activation and blood flow simultaneously and contribute to the heterogeneity of NVC investigations. Aging has its own detrimental effects on cerebral hemodynamics (124) and increasing age is associated with increasing prevalence of comorbid conditions such as hypertension (125) and obesity (126). Arterial vascular pathology produces multiple secondary effects, lowering capillary density, disrupting the BBB, damaging the endothelia, reducing contractility, increasing pulsatility and compromising retrograde propagation (122). In some of these cases, CO_2_-enriched air may trigger vascular steal phenomena which necessitates evaluation of the global vascular haemodynamics. Other variables such as time of day, level of arousal, alcohol, caffeine, exercise, menstrual phase and medications also affect measurements (127). Age, sex and body-mass index influence cardiac output distribution to the brain but complete correlation between neurocognitive and neurovascular ageing is not given and reduced CBF in the elderly does not seem to result from age-related decreases in cardiac output (128). Further, age likely affects glia and neurons differently and changes may be driven more by one group of cells.

Lastly, as stated above, each modality has distinct characteristics with regards to temporal and spatial resolution. With regards to temporal resolution, ultrasound has the highest and PET the lowest. With regards to spatial resolution, with some variation, fMRI and ASL are likely superior. However, a typical fMRI pixel size of around 3-4 mm is still far from the approximately 200 µm at which some mechanisms have been described for NO-mediation of NVC between neurons and arterioles in the rat hippocampus (71). The anatomical substrate is present since both neurons and smooth muscle cells can co-inhabit a space of this size (129). While higher-field systems may provide greater spatial resolution they are still no substitute for the insights which invasive animal studies can provide.

## Conclusions and Future Research Trajectories

The cellular mechanisms regulating NVC are complex and still incompletely understood. Each modality used to measure NVC in humans has its limitations, and the multiple confounding variables need to be considered in the population of interest. Despite of these limitations, there is converging evidence for an independent effect of the T2DM-state on NVC with cognitive decline as a possible progressing clinical correlate. Potentially, all steps of the NVC-cascade may be affected by separate diabetes-induces changes and currently it is impossible to discern which are clinically relevant. Further, how the induced pathological changes precisely affect measurements of the discussed modalities needs clarification.

Early detection of impaired NVC in T2DM patients could represent an opportunity for initiation of preventive treatment before irreversible damage occurs, especially since it is plausible that novel therapeutics may directly or indirectly involve NVC. Future studies could explore subgroups of T2DM where specific aspects of CBF control may be compromised such as those with autonomic neuropathy. NVC effects of medications such as pioglitazone and GLP-1 receptor agonists with effects on insulin sensitivity and low-activity inflammation commonly used in diabetes also need further exploration.

## Author Contributions

MB wrote the first draft of the manuscript. EP, SM and HS wrote sections of the manuscript. All authors contributed to conception and design of the review, manuscript revision, read, and approved the submitted version.

## Funding

HS holds a 5-year professorship in precision medicine at the Faculty of Health Sciences and Medicine, University of Copenhagen which is sponsored by the Lundbeck Foundation (Grant Nr. R186-2015-2138).

## Conflict of Interest

MB has received honoraria as a speaker for Autonomic Technologies Inc. HS has received honoraria as speaker from Sanofi Genzyme, Denmark and Novartis, Denmark, as consultant from Sanofi Genzyme, Denmark, Lophora, Denmark, and Lundbeck AS, Denmark, and as editor-in-chief (Neuroimage Clinical) and senior editor (NeuroImage) from Elsevier Publishers, Amsterdam, The Netherlands. He has received royalties as book editor from Springer Publishers, Stuttgart, Germany and from Gyldendal Publishers, Copenhagen, Denmark. SM: *Advisory boards:* AstraZeneca; Boehringer Ingelheim; Eli Lilly; Merck Sharp & Dohme; Novo Nordisk; Sanofi, Bayer. *Lecture fees:* AstraZeneca; Boehringer Ingelheim; Merck Sharp & Dohme; Novo Nordisk; Sanofi. *Research Grant Recipient*: Novo Nordisk, Boehringer-Ingelheim.

The remaining authors declare that the research was conducted in the absence of any commercial or financial relationships that could be construed as a potential conflict of interest.

## Publisher’s Note

All claims expressed in this article are solely those of the authors and do not necessarily represent those of their affiliated organizations, or those of the publisher, the editors and the reviewers. Any product that may be evaluated in this article, or claim that may be made by its manufacturer, is not guaranteed or endorsed by the publisher.
